# ARRDC1 inhibits the replication of Semliki Forest virus by regulating the ubiquitination and degradation of viral nsP4

**DOI:** 10.1128/jvi.00977-25

**Published:** 2025-08-18

**Authors:** Qinyu Peng, Xiaoyi Yang, Cancan Chen, Junfang He, Yingan Liang, Xiaotong Luo, Changbai Huang, Wenbi Wu, Ping Zhang, Chao Liu

**Affiliations:** 1 Key Laboratory of Tropical Diseases Control (Sun Yat-sen University), Ministry of Education, Guangzhou, Guangdong, China; 2 Department of Immunology and Microbiology, Zhongshan School of Medicine, Sun Yat-sen University 26469 , Guangzhou, Guangdong, China; 3 Department of Pathology, The First Affiliated Hospital of Sun Yat-sen University 71068 , Guangzhou, Guangdong, China; 4 State Key Laboratory of Biocontrol, School of Life Sciences, Sun Yat-sen University 26469 , Guangzhou, Guangdong, China; University of North Carolina at Chapel Hill, Chapel Hill, North Carolina, USA

**Keywords:** SFV, ARRDC1, restriction factor, ubiquitination, nsP4

## Abstract

Alphavirus infection can result in a spectrum of clinical manifestations in the host, including fever, rash, arthritis, and even symptoms of encephalitis, thereby posing a severe threat to global public health security. In this study, we explored the role of arrestin domain-containing protein 1 (ARRDC1) in the replication of Semliki Forest virus (SFV), an important member of alphaviruses, by siRNA-based knockdown or CRISPR/Cas9-mediated knockout techniques. SFV replication levels are significantly increased by knockdown or knockout of ARRDC1 in multiple cell lines and inhibited by trans-complementation with ARRDC1. Our data further revealed that ARRDC1 affects the early RNA replication stage of SFV. The antiviral effect of ARRDC1 is dependent on its cell plasma membrane localization and ubiquitin ligase binding motif. Mechanistically, ARRDC1 binds to viral nonstructural protein 4 (nsP4) and facilitates its degradation by the ubiquitination pathway, thereby blocking the replication of SFV. In summary, this work identifies ARRDC1 as a novel restriction factor of SFV, potentially advancing the development of novel strategies against alphavirus infection.

**IMPORTANCE:**

Semliki Forest virus (SFV) belongs to the *Alphavirus* genus in the *Togaviridae* family and can cause a spectrum of clinical manifestations in the host, including fever, rash, arthritis, and even symptoms of encephalitis. Here, we reveal that ARRDC1 is a novel restriction factor for SFV in multiple cell lines, which relies on its cell plasma membrane localization and ubiquitin ligase binding motif. Interestingly, we further provide evidence that upon interacting with SFV nsP4, ARRDC1 mediates the degradation of nsP4 via the ubiquitination pathway, thereby inhibiting viral replication. Our study elucidates a new antiviral mechanism of ARRDC1 by mediating the ubiquitination and degradation of viral protein, which is of great significance for further understanding the pathogenesis of alphaviruses and the development of potential antiviral strategies.

## INTRODUCTION

Alphaviruses are a group of single-stranded positive-sense RNA viruses that belong to the *Togaviridae* family. They are primarily transmitted by arthropod vectors and can infect both vertebrates and invertebrates (1, 2). These viruses can cause a range of symptoms in humans, including fever, joint pain, and even encephalitis (3, 4), thus posing a significant burden on the global public health system. Alphaviruses are divided into Old World and New World alphaviruses based on their geographical distribution (2). Among them, Semliki Forest virus (SFV), Sindbis virus (SINV), chikungunya virus (CHIKV), and Ross River virus (RRV) are categorized as Old World alphaviruses, while western equine encephalitis virus (WEEV), eastern equine encephalitis virus (EEEV), and Venezuelan equine encephalitis virus (VEEV) are classified as New World alphaviruses (2). Old World alphaviruses mainly cause arthritis and musculoskeletal disorders, whereas New World alphaviruses primarily lead to meningitis and encephalitis, often accompanied by long-term neurasthenic sequelae (5).

Among the Old World alphaviruses, SFV is a well-known example. It is mainly transmitted by mosquitoes and can cause a spectrum of clinical manifestations in the host, including fever, rash, arthritis, and encephalitis (5). The SFV genome comprises two separate open reading frames (ORFs). The first ORF is translated into nonstructural (NS) polyproteins P123 and P1234, which are further processed by nsP2 into nsP1, nsP2, nsP3, and nsP4 proteins (6). These nsPs are subsequently transferred to the cell plasma membrane, where they form spherules and facilitate early RNA replication (7, 8). The second ORF of the SFV genome encodes structural proteins, including capsid protein and three envelope glycoproteins, which are essential for virus assembly and maturation. The envelope glycoproteins play particularly key roles in viral attachment and entry into host cells, initiating a new round of infection (6, 9–11).

A variety of host proteins have been identified as antiviral factors combating alphaviruses. For example, DDX39A directly binds to viral RNA, thereby controlling alphavirus infection (12). ZAP restricts alphavirus replication by binding viral RNA genomes to block translation and induce RNA degradation (13, 14). Moreover, TIPARP interacts with the Getah virus (GETV) E2 glycoprotein, inducing K48-linked ubiquitination and subsequent proteasome degradation (15). Nonetheless, there are still a number of restriction factors of alphaviruses that remain to be identified.

Arrestin domain-containing protein 1 (ARRDC1) is a member of the α-arrestins family, also known as arrestin-related trafficking adaptors (ARTs), which are conserved in eukaryotes (16). ARRDC1 possesses a Pro-Pro-x-Tyr (PPxY) motif that interacts with HECT E3 ubiquitin ligases, facilitating the ubiquitination and degradation of membrane proteins. The C-terminal domain of ARRDC1 includes two PPxY motifs (Pro-Pro-Glu-Tyr, PPEY, and Pro-Pro-Ser-Tyr, PPSY), while its N-terminal arrestin domain aids in anchoring to the inner membrane (17, 18). ARRDC1 can mediate the release of ARRDC1-mediated microvesicles (ARMMs) from the cell plasma membranes through the endosomal sorting complexes required for transport (ESCRT) pathway. This process involves interactions with two key components: Tumor susceptibility gene 101 (TSG101) and Vacuolar protein sorting-associated 4 (VPS4) (18). The process by which the Gag protein of human immunodeficiency virus type 1 (HIV-1) utilizes TSG101 to facilitate the release of virions is similar to that of ARMMs (19), suggesting a potential role for ARRDC1 in other virus infections. So far, several members of the α-arrestins family, such as ARRDC1, ARRDC2, ARRDC3, and ARRDC4, have been shown to participate in virus replication. ARRDC1, ARRDC2, and ARRDC3 inhibit the budding of Moloney murine leukemia virus (MMLV) through interactions with HECT ubiquitin ligases and the ESCRT pathway (17). Knockdown of ARRDC3 reduces the sensitivity to human papillomavirus (HPV) infection but enhances the replication of enterovirus D68 (EV-D68) (20, 21). ARRDC4 activates the innate immune pathway to suppress EV71 replication (22). Nonetheless, whether ARRDC1 plays a role in the infection of alphavirus remains unclear.

In this study, we investigated the role of ARRDC1 in SFV replication by generating ARRDC1-knockdown or -knockout cell clones. We discovered that ARRDC1 has a significant antiviral effect on SFV replication, depending on its cell plasma membrane localization and ubiquitin ligase binding motif. Furthermore, we demonstrated that ARRDC1, upon binding to SFV nsP4, promotes the degradation of nsP4 through the ubiquitination pathway, which in turn suppresses the replication of SFV. Hence, our work illustrated the mechanism by which SFV nsP4 is degraded and how ARRDC1 inhibits SFV replication, thereby proposing a potential antiviral strategy for alphaviruses.

## RESULTS

### ARRDC1 plays an inhibitory role in SFV replication

**Fig 1:**
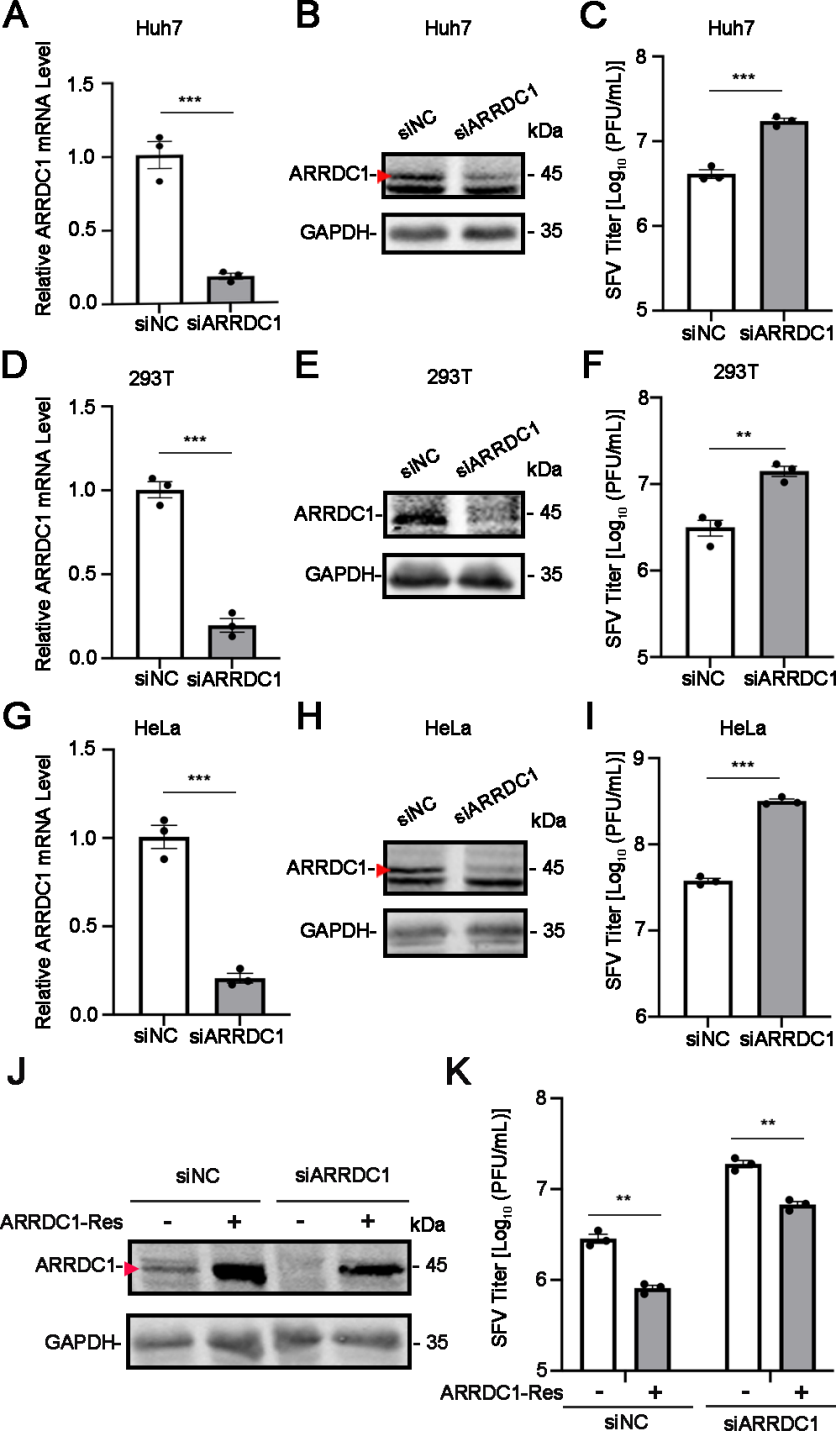
The replication of SFV is increased in ARRDC1-knockdown cells. (**A–C**) SFV replication levels in ARRDC1-knockdown Huh7 cells. Huh7 cells were transfected with siNC or siARRDC1. At 36 h post-transfection (p.t.), the cells were infected with SFV at a multiplicity of infection (MOI) of 0.01. Then, the cells were harvested at 12 h post-infection (p.i.) for the measurement of *ARRDC1* mRNA levels (**A**) and protein levels (**B**). The supernatants were collected for plaque assay (**C**). (**D–F**) SFV replication levels in ARRDC1-knockdown 293T cells. 293T cells were transfected with siNC or siARRDC1, followed by the infection of SFV at an MOI of 0.01. Then, the cells were harvested at 12 h p.i. for the measurement of *ARRDC1* mRNA levels (**D**) and protein levels (**E**). The supernatants were collected for plaque assay (**F**). (**G–I**) SFV replication levels in ARRDC1-knockdown HeLa cells. HeLa cells were transfected with siNC or siARRDC1. At 36 h p.i., the cells were infected with SFV at an MOI of 1. Then, the cells were harvested at 12 h p.i. for the measurement of *ARRDC1* mRNA levels (**G**) and protein levels (**H**). The supernatants were collected for plaque assay (**I**). (**J–K**) The effect of ARRDC1 restoration on SFV replication levels. The ARRDC1-knockdown HeLa cells were transfected with vector or ARRDC1-expressing plasmid, followed by SFV infection at an MOI of 1. At 12 h p.i., cells and supernatants were harvested for western blot using anti-ARRDC1 (**J**) or plaque assay (**K**). Human *GAPDH* mRNA level was detected as an internal control for quantitative reverse transcription PCR (qRT-PCR). GAPDH was probed as an internal control for western blot (B, E, H, and J). Representative images of three independent experiments are shown. Data are shown as mean ± SEM from at least three independent experiments. Statistical significance was determined using an unpaired two-tailed *t*-test. ns, no statistical significance; **, *P* < 0.01; ***, *P* < 0.001.

To explore the role of ARRDC1 in SFV infection, we first detected the effect of ARRDC1 on SFV replication by silencing ARRDC1 in Huh7 cells. As shown in Fig. 1A and B, the mRNA and protein levels of ARRDC1 in siARRDC1-transfected cells were significantly reduced compared to siNC-transfected cells, demonstrating that the knockdown of ARRDC1 was effective. Subsequently, both the control cells and ARRDC1-knockdown cells were infected with SFV to detect the viral replication levels. Plaque assay revealed that the knockdown of ARRDC1 resulted in a significant increase of SFV titer (Fig. 1C). A similar phenomenon was also observed in 293T and HeLa cells (Fig. 1D through I), suggesting that ARRDC1 can inhibit SFV replication. To rule out potential off-target effects, the rescue mutant of ARRDC1 was transfected into the ARRDC1-knockdown HeLa cells. The data showed that levels of ARRDC1 protein and viral titer were all restored in ARRDC1-rescued cells (Fig. 1J and K).

**Fig 2:**
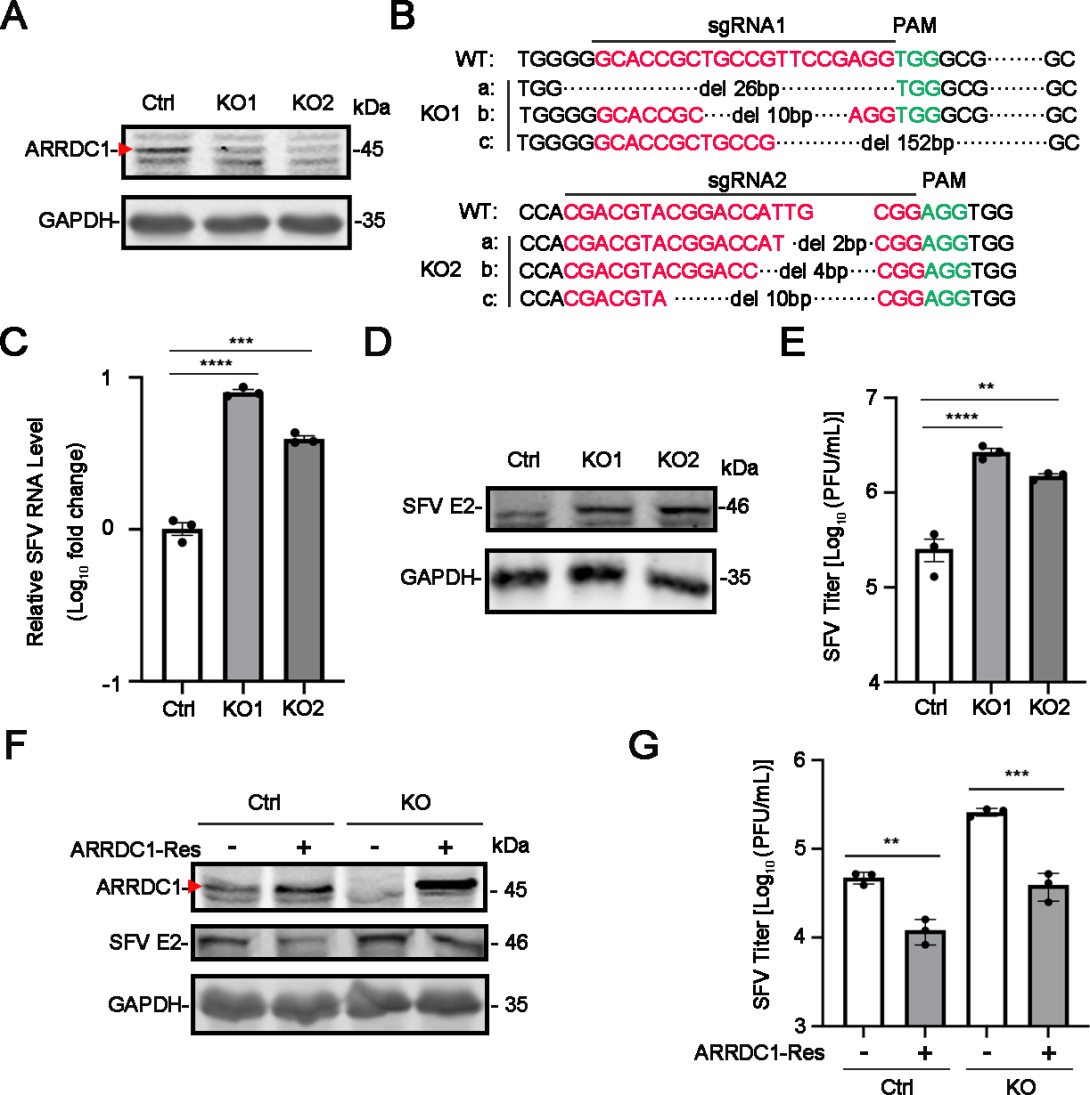
SFV infection is increased in ARRDC1-KO cells. Confirmation of ARRDC1 knockout efficiency. (A) Knockout efficiency of two ARRDC1-KO clones (ARRDC1^KO1^ and ARRDC1^KO2^) in A549 cells was validated by western blot using anti-ARRDC1 antibody. (B) DNA sequencing analysis of ARRDC1-KO cells. (C–E) Effect of ARRDC1 knockout on SFV replication levels. Intracellular RNA levels (C), E2 protein levels (D), and titers (E) in SFV-infected control and ARRDC1-KO cells were measured by qRT-PCR, western blot, and plaque assay, respectively. Human *GAPDH* mRNA level was detected as an internal control for qRT-PCR. (F–G) The effect of ARRDC1 restoration on SFV replication levels. The control and ARRDC1-KO A549 cells transfected with vector or ARRDC1-expressing plasmid were infected with SFV at MOI 1. At 12 h p.i., cells and supernatants were harvested for western blot using anti-ARRDC1 and anti-SFV E2 antibodies (F) or plaque assay (G). Human *GAPDH* mRNA level was detected as an internal control for qRT-PCR. GAPDH was probed as an internal control for western blot (A, D, and F). Representative images of three independent experiments are shown. Data are shown as mean ± SEM from at least three independent experiments. Statistical significance was determined using one-way analysis of variance (ANOVA) with Dunnett’s multiple comparison test (C and E) or unpaired two-tailed *t*-test (G). ns, no statistical significance; **, *P* < 0.01; ***, *P* < 0.001; ****, *P* < 0.0001.

Furthermore, we utilized the CRISPR/Cas9 gene-editing technique to generate two independent ARRDC1-knockout (KO) A549 cells. Western blot and DNA sequencing experiments were used to detect the knockout efficiency of ARRDC1. As shown in Fig. 2A and B, ARRDC1 was successfully knocked out in ARRDC1-KO cells. Since the ARRDC1 protein is difficult to detect and the high antibody concentration (1:500 dilution) may lead to elevated background signals, the residual bands observed in western blot were likely nonspecific. Then, the control and ARRDC1-KO cells were infected with SFV for a viral replication level assay. As shown in Fig. 2C to E, viral RNA, E2 protein, and titer levels in the ARRDC1-KO cells were significantly increased compared to the control cells. Moreover, upon transfection of a rescue mutant of ARRDC1 into the KO cells, we observed a significant decrease in viral RNA, E2 protein, and titer levels (Fig. 2F and G). Taken together, these data demonstrated that ARRDC1 inhibits the replication of SFV.

### ARRDC1 affects the replication of multiple positive-strand RNA viruses

**Fig 3:**
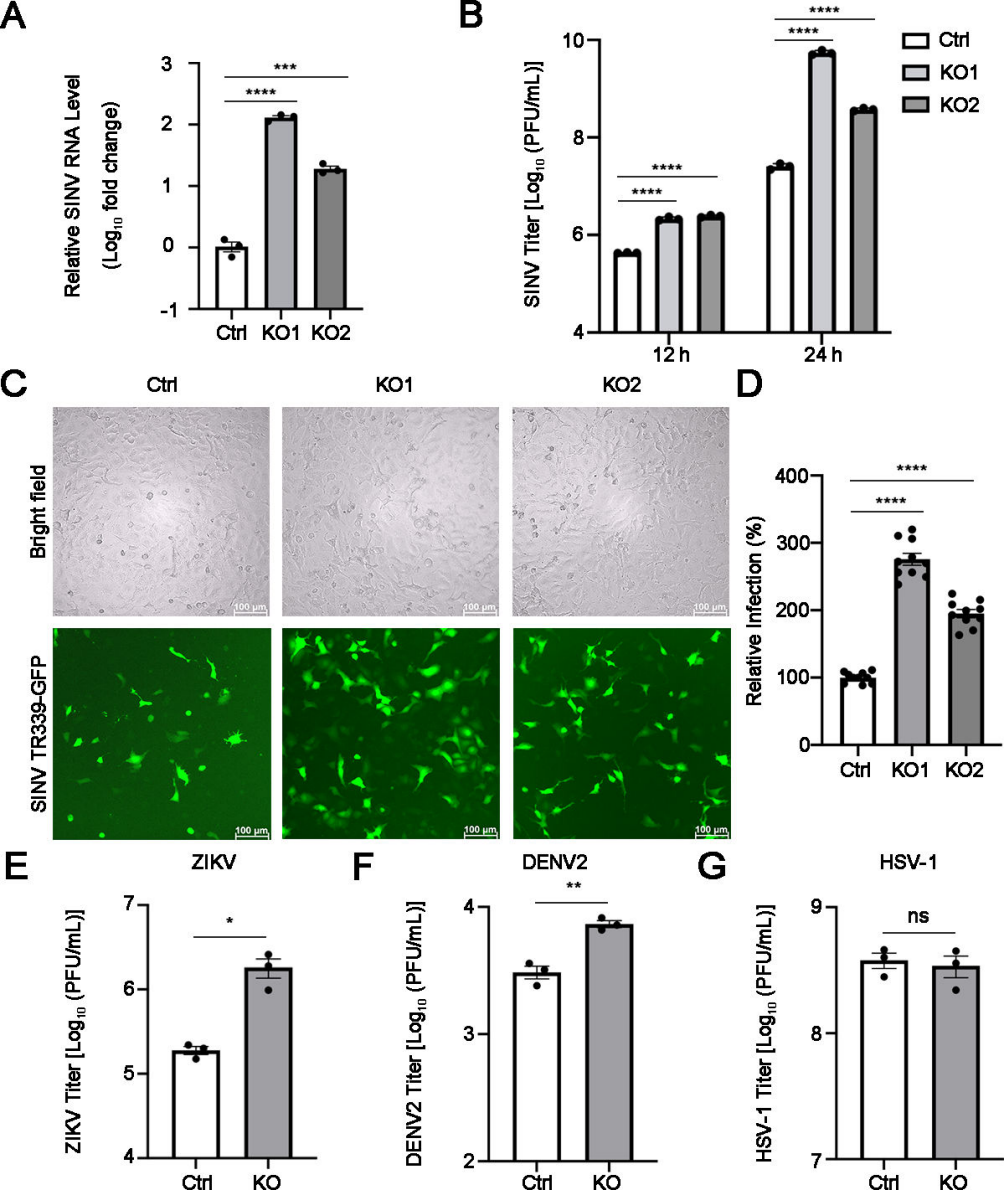
ARRDC1 affects the replication of multiple positive-strand RNA viruses. (**A–B**) Effect of ARRDC1 knockout on SINV replication levels. Intracellular RNA levels of SINV-infected control and ARRDC1-KO cells (MOI = 1) at 24 h p.i. were measured by qRT-PCR (**A**). Human *GAPDH* mRNA level was detected as an internal control for qRT-PCR. Titers in SINV-infected control and ARRDC1-KO cells (MOI = 1) at 12 and 24 h p.i. were measured by plaque assay, respectively (**B**). (**C–D**) Impact of ARRDC1 knockout on SINV infectivity. The infectivity of SINV TR339-GFP-infected control and ARRDC1-KO cells (MOI = 1) at 24 h p.i. was observed using a fluorescence microscope DMi8. The green fluorescence signal represents an SINV TR339-GFP-infected cell (**C**), which was statistically analyzed using Image J software (**D**). (**E–G**) Effect of ARRDC1 knockout on the replication of Zika virus (ZIKV), dengue virus serotype 2 (DENV2), or herpes simplex virus type 1 (HSV-1). Titers in ZIKV- (**E**), DENV2- (**F**), and HSV-1-infected control and ARRDC1-KO cells (MOI = 1) at 24 h p.i. were measured by plaque assay. Human *GAPDH* mRNA level was detected as an internal control for qRT-PCR. Representative images of three independent experiments are shown. Data are shown as mean ± SEM from at least three independent experiments. Statistical significance was determined using ANOVA with Dunnett’s multiple comparison test (A, B, and D) or unpaired two-tailed *t*-test (**E–G**). ns, no statistical significance; ***, *P* < 0.001; ****, *P* < 0.0001.

To investigate whether ARRDC1 affects other alphavirus replication, we infected the control cells and ARRDC1-KO cells with SINV (multiplicity of infection [MOI] = 1), another member of the Old World alphavirus (23). At 24 h post-infection (p.i.), SINV RNA levels in ARRDC1-KO cells were increased by about 125-fold compared to the control cells (Fig. 3A). The virus titers in ARRDC1-KO cells were increased by about 5-fold at 12 h p.i. and 242-fold at 24 h p.i., respectively (Fig. 3B). The SINV-GFP-infected cells were observed by fluorescence microscope at 24 h p.i. The results showed that the infection rate of SINV in the ARRDC1-KO cells was enhanced by about 3-fold compared to the control cells (Fig. 3C and D), suggesting that the replication levels of SINV in ARRDC1-KO cells were significantly higher than those in the control cells. The above results indicated that ARRDC1 also inhibits the replication of SINV.

In addition, we investigated whether ARRDC1 regulates the replication of another two positive-strand RNA viruses (Zika virus, ZIKV, and dengue virus, DENV) and one DNA virus (herpes simplex virus type 1, HSV-1). The data showed that knockout of ARRDC1 enhanced the replication of both ZIKV and DENV (Fig. 3E and F), but not HSV-1 (Fig. 3G), implying that ARRDC1 suppresses the replication of multiple positive-strand RNA viruses tested, but not the DNA virus tested.

### ARRDC1 inhibits SFV RNA replication in the early stage

**Fig 4:**
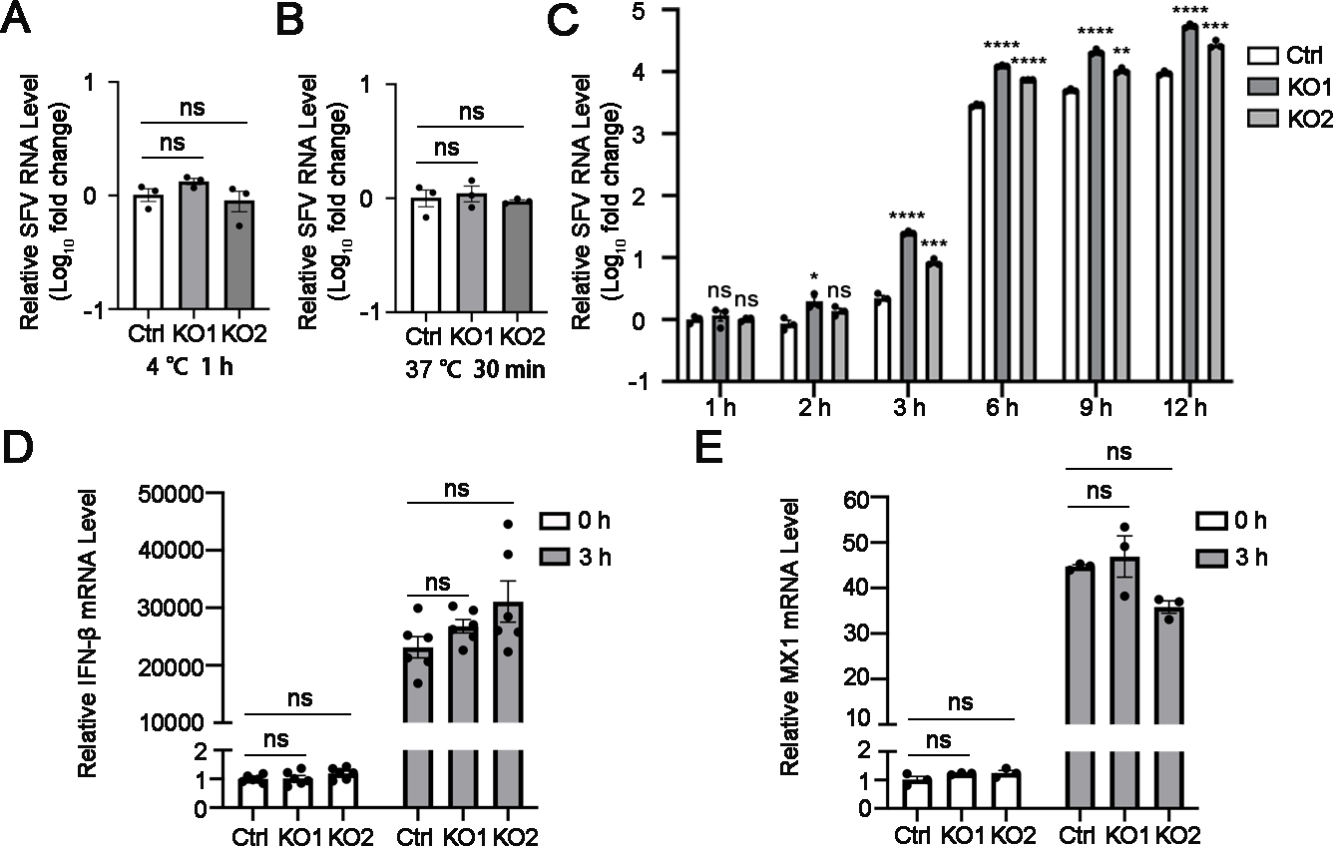
ARRDC1 inhibits SFV RNA replication in the early stage. (**A–B**) Viral entry assay. The control and ARRDC1-KO A549 cells were inoculated with SFV (MOI =1) at 4°C for 1 h or 37°C for 30 min. The cells were harvested for qRT-PCR to detect the levels of attached virions (**A**) or internalized virions (**B**). (**C**) Effect of ARRDC1 on SFV RNA replication. Intracellular RNA levels of SFV-infected control and ARRDC1-KO cells (MOI = 1) at 1, 2, 3, 6, 9, and 12 h p.i. were measured by qRT-PCR, respectively. (**D–E**) Impact of ARRDC1 on *IFN* mRNA levels. The control and ARRDC1-KO cells were transfected with 1 μg/mL poly (I:C) for 3 h. The cells were collected for the detection of *IFN-β* (**D**) and *MX1* (**E**) mRNA levels. (**A–E**) Human *GAPDH* mRNA level was detected as an internal control for qRT-PCR. Data are shown as mean ± SEM from at least three independent experiments. Statistical significance was determined using ANOVA with Dunnett’s multiple comparison test (**A–E**). ns, no statistical significance; *, *P* < 0.05; **, *P* < 0.01; ***, *P* < 0.001; ****, *P* < 0.0001.

To explore the mechanism by which ARRDC1 inhibits SFV infection, we first examined whether ARRDC1 affects the adsorption and endocytosis of SFV. Cells were inoculated with viruses at 4°C for 1 h to detect virion attachment or at 37°C for 30 min to analyze virion internalization. Total RNAs were extracted for quantitative reverse transcription PCR (qRT-PCR) to quantify the RNA levels of attached or internalized virions. Notably, the RNA levels of SFV in both control and ARRDC1-KO cells were comparable (Fig. 4A and B), indicating that ARRDC1 does not modulate the adsorption and endocytosis stages of SFV.

Next, to detect whether ARRDC1 regulates the viral replication process, the SFV RNA levels in the control and ARRDC1-KO cells were compared at different time points. At 1 and 2 h p.i., SFV RNA levels in all groups were comparable; however, a significant increase in viral RNA levels was observed in the ARRDC1-KO cells at 3 h p.i. (Fig. 4C), suggesting that ARRDC1 plays an antiviral role in the early stage of SFV RNA replication.

Considering that ARRDC4 has been reported to regulate the innate immune signaling pathway during EV71 infection (22), and the interferon (IFN) signaling pathway is also rapidly activated in the early stage of SFV infection, we speculated that ARRDC1 might suppress the SFV early stage by modulating the innate immune response. Thus, we treated the control and ARRDC1-KO cells with poly (I:C) and measured the mRNA levels of the IFN pathway. The mRNA levels of *IFN-β* and *MX1* were comparable in both control and ARRDC1-KO cells (Fig. 4D and E), suggesting that the inhibitory effect of ARRDC1 on SFV replication is independent of the innate immune pathway.

### The antiviral function of ARRDC1 depends on its cell plasma membrane localization and ubiquitin ligase binding motif

**Fig 5:**
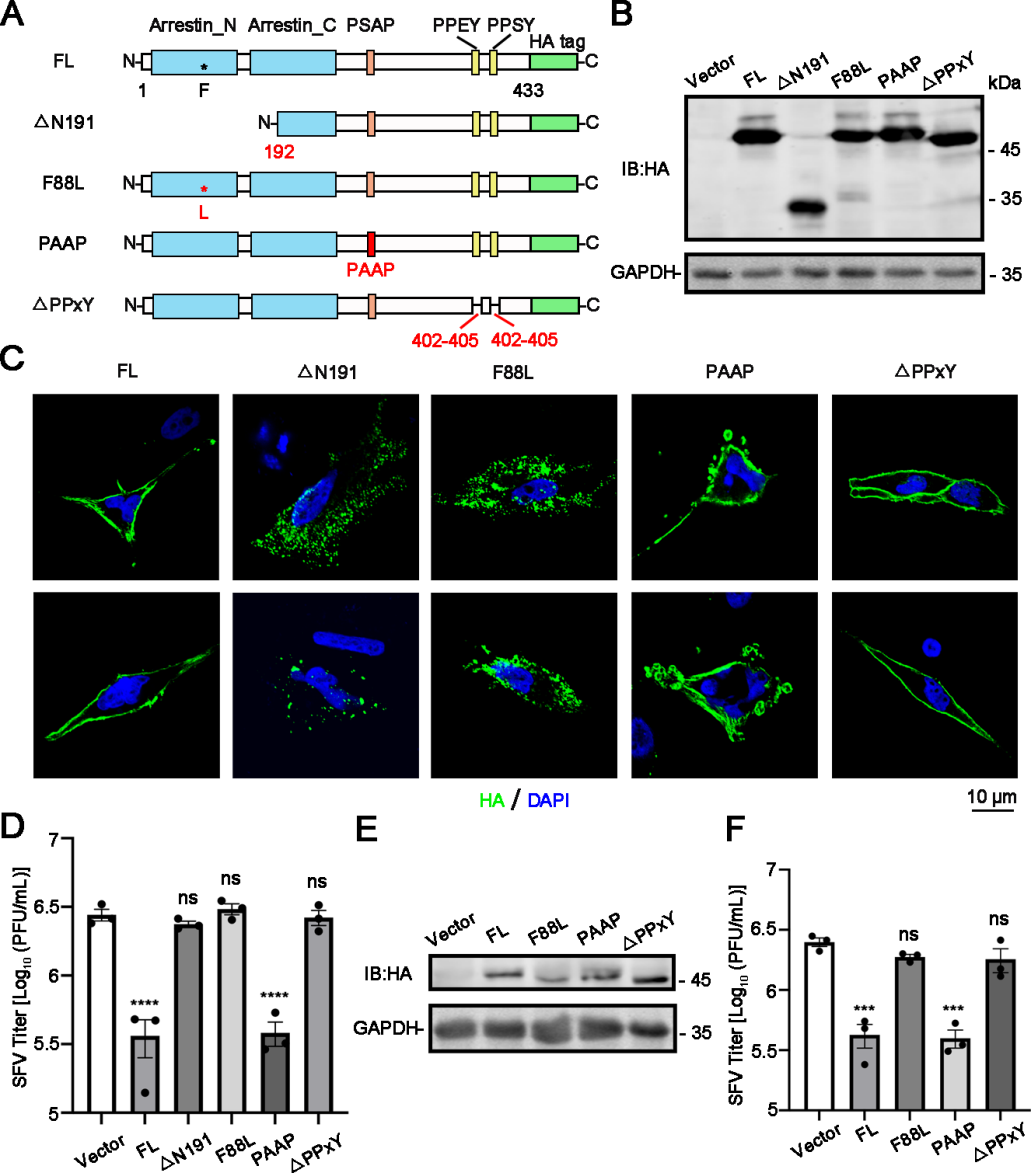
The cell plasma membrane localization and ubiquitin ligase binding motif of ARRDC1 are required for its antiviral function. Schematic representation of full-length and mutated ARRDC1-HA constructs. (**B**) Detection of ARRDC1 expression levels. Protein levels of full-length and mutated ARRDC1-HA constructs (△N191, F88L, PAAP, and △PPxY) in 293T cells were detected by western blot. (**C**) Subcellular distributions of ARRDC1. The plasmids expressing full-length ARRDC1-HA and its mutants (△N191, F88L, PAAP, and △PPxY) were transfected into A549 cells for 24 h. The cells were harvested for immunostaining using anti-HA antibody (green). DAPI stains the nuclei (blue). Cell images were captured by a Confocal Microscope (Scale bars, 10 μm). (**D–F**) SFV replication levels. The plasmids expressing full-length ARRDC1-HA and its mutants (△N191, F88L, PAAP, and △PPxY) were transfected into 293T cells for 24 h, followed by SFV infection at an MOI of 0.01. At 12 h p.i., supernatants were harvested for plaque assay (**D**). The plasmids expressing full-length ARRDC1-HA and its mutants (F88L, PAAP, and △PPxY) were transfected into ARRDC1-KO A549 cells for 24 h, followed by SFV infection (MOI = 1). At 12 h p.i., cells and supernatants were harvested for western blot (**E**) and plaque assay (**F**). Human *GAPDH* mRNA level was detected as an internal control for qRT-PCR. GAPDH was probed as an internal control for western blot (**B and E**). Representative images of three independent experiments are shown. Data are shown as mean ± SEM from at least three independent experiments. Statistical significance was determined using one-way ANOVA with Dunnett’s multiple comparison test (**D and F**). ns, no statistical significance; **, *P* < 0.01; ***, *P* < 0.001.

ARRDC1 is composed of three domains: a cell plasma membrane localization domain, a ubiquitin ligase binding domain, and an ESCRT component binding domain. The amino acids (aa) 88, 180, and 191 of ARRDC1, particularly aa 88, are crucial aa determining its cell plasma membrane localization (18). In order to determine the key domain and amino acids of ARRDC1 mediating its antiviral effect, we constructed four ARRDC1 mutants (Fig. 5A): ARRDC1-△N191 and ARRDC1-F88L mutants which cannot localize to plasma membrane, ARRDC1-PAAP mutant which cannot interact with TSG101, and ARRDC1-△PPxY mutant which lacks both PPEY and PPSY motifs and does not interact with ubiquitin ligases and participate in the ubiquitination of substrate proteins (17, 18).

Subsequently, we detected the impact of these ARRDC1 mutants on SFV replication. Plasmids expressing full-length ARRDC1 or its mutants were transfected into 293T cells. At 24 h post-transfection (p.t.), cells were lysed for western blot to validate the expression levels of ARRDC1 constructs (Fig. 5B). The localization of these mutants in A549 cells was examined by immunofluorescence assay. As expected, full-length ARRDC1, together with PAAP and △PPxY mutants, was localized on the cell plasma membrane. In contrast, the △N191 and F88L mutants were dispersed in the cytoplasm (Fig. 5C). At 24 h p.t., cells were infected with SFV, and the supernatant was collected at 12 h p.i. for plaque assay. The results revealed that viral titers in 293T cells expressing full-length ARRDC1 or the PAAP mutant were significantly decreased compared to the control cells, while titers in cells expressing the △N191, F88L, or △PPxY mutants showed no significant change (Fig. 5D), indicating that both the cell plasma membrane localization and ubiquitin ligase binding domains of ARRDC1 are crucial for SFV replication.

The full-length ARRDC1 and its mutants were also introduced into ARRDC1-KO cells. Due to the poor expression of the △N191 mutant in ARRDC1-KO A549 cells, we focused on the F88L mutant to verify the role of the membrane localization domain in the antiviral capability of ARRDC1. Expression levels of each mutant in the ARRDC1-KO cells were comparable (Fig. 5E). In the ARRDC1-KO cells, complementation of full-length ARRDC1 or the PAAP mutant, but not the F88L or △PPxY mutant, markedly reduced the viral titers compared to the vector-transfected cells (Fig. 5F), suggesting that the inhibitory effect of ARRDC1 on SFV replication depends on its cell plasma membrane localization and ubiquitin ligase binding ability.

### ARRDC1 mediates the degradation of nsP4 through the ubiquitination pathway

**Fig 6:**
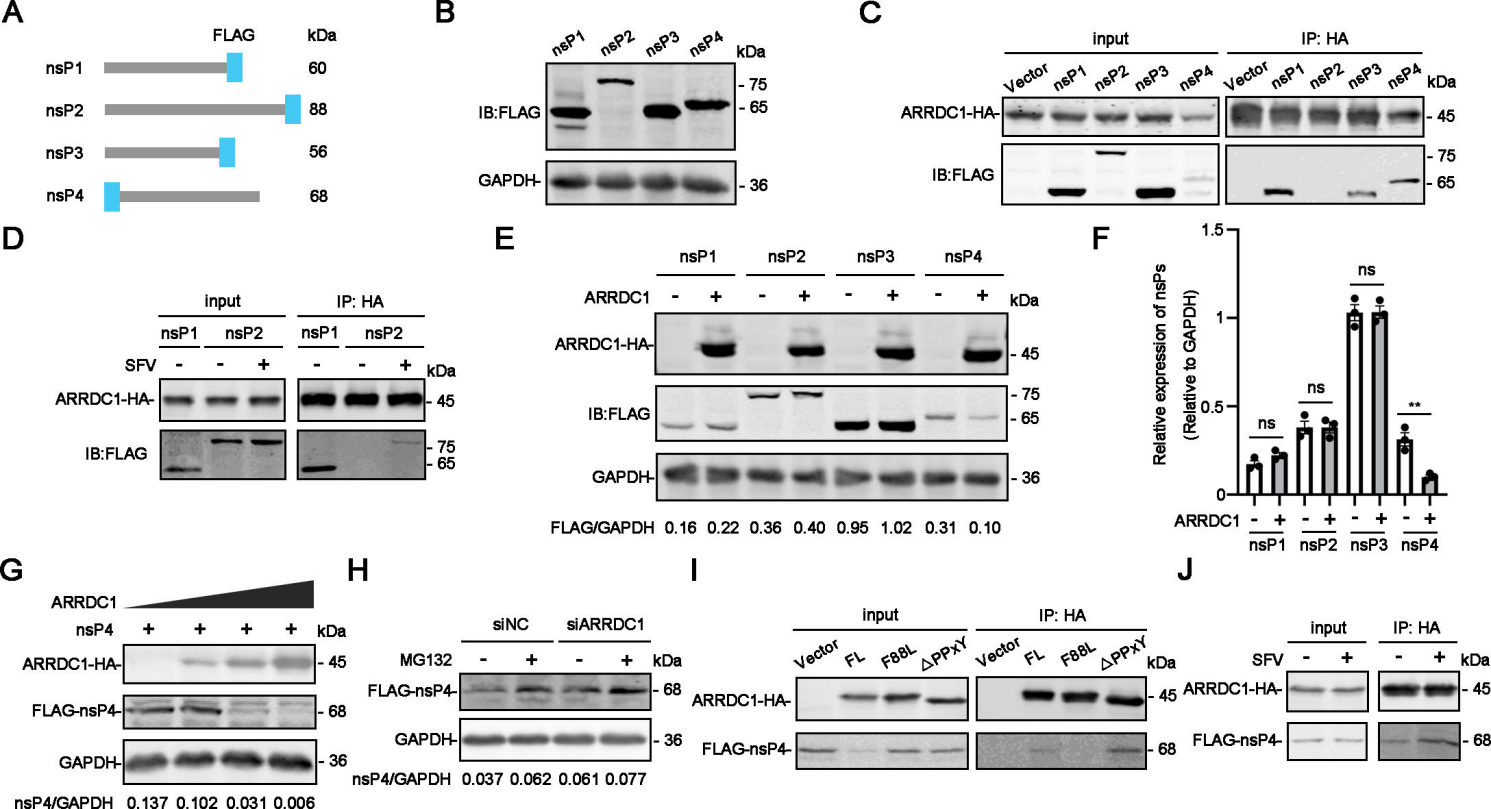
ARRDC1 mediates the degradation of nsP4 through ubiquitination modification. Schematic illustration of constructs expressing FLAG-tagged SFV nsP1, nsP2, nsP3, and nsP4. (**B**) Expression levels of SFV nsP constructs. FLAG-tagged SFV nsPs expression constructs were transfected into 293T cells for western blot using anti-FLAG antibody. (**C**) Co-IP assay of ARRDC1 and SFV nsPs. The plasmids expressing ARRDC1-HA and FLAG-tagged SFV nsPs were co-transfected into 293T cells for an immunoprecipitation assay using anti-HA agarose beads. The immunoprecipitated samples were then detected by western blot using anti-HA and anti-FLAG antibodies. (**D**) Co-IP assay of ARRDC1 and nsP2 during SFV infection. The plasmids expressing ARRDC1-HA and FLAG-tagged SFV nsP2 (nsP1-FLAG as a positive control) were co-transfected into 293T cells for 24 h, followed by SFV infection. At 12 h p.i., cells were harvested for immunoprecipitation assay using anti-HA agarose beads. The immunoprecipitated samples were then detected by western blot using anti-HA and anti-FLAG antibodies. (**E–F**) Impact of ARRDC1 on the expression levels of SFV nsPs. 293T cells were co-transfected with plasmids expressing ARRDC1-HA and FLAG-tagged SFV nsPs, respectively. At 24 h p.t., the expression levels of ARRDC1 and SFV nsPs were detected using anti-HA and anti-FLAG antibodies (**E**). Quantitative analysis of the nsP4/GAPDH ratio from three independent replicates was shown (**F**). (**G**) Effect of ARRDC1 on SFV nsP4 levels. 293T cells were co-transfected with 400 ng of FLAG-tagged SFV nsP4- and different amounts of ARRDC1-HA-expressing plasmids (0, 200, 400, or 800 ng) for western blot using anti-HA and anti-FLAG antibodies. (**H**) Impact of MG132 on nsP4 degradation. The control and ARRDC1-knockdown 293T cells were transfected with a FLAG-tagged nsP4 expressing plasmid, followed by the treatment with DMSO or 10 μM MG132 for 1 h. Subsequently, the cell extracts were collected for western blot analysis with anti-FLAG antibody. (**I**) Co-IP assay of ARRDC1 mutants and SFV nsP4. The plasmids expressing ARRDC1-HA mutants (F88L and △PPxY, full-length ARRDC1-HA as a positive control) and FLAG-tagged SFV nsP4 were co-transfected into 293T cells for an immunoprecipitation assay using anti-HA agarose beads. (**J**) Co-IP assay of ARRDC1 and nsP4 during SFV infection. ARRDC1-HA- and FLAG-tagged SFV nsP4-expressing plasmids were co-transfected into 293T cells for 24  h, followed by SFV infection. At 12 h p.i., cells were harvested for immunoprecipitation assay using anti-HA agarose beads. The immunoprecipitated samples were then detected by western blot using anti-HA and anti-FLAG antibodies (**I and J**). GAPDH was probed as an internal control for western blot (**B–J**). Representative images of three independent experiments are shown. Statistical significance was determined using an unpaired two-tailed *t*-test. ns, no statistical significance; **, *P* < 0.01.

Previous studies reported that ARRDC1 can interact with various membrane proteins and promotes their degradation through the ubiquitination pathway (24). SFV nsP1 serves as the membrane-anchored subunit of the replication complex, while other nsPs (nsP2, nsP3, and nsP4) are recruited to the membrane through their association with nsP1 in the form of polyprotein precursors. This process is crucial for early RNA replication (25). Therefore, we hypothesized that ARRDC1 might inhibit SFV replication by promoting the degradation of viral nsPs via its ubiquitin ligase binding ability. To test this hypothesis, we first detected whether ARRDC1 interacts with SFV nsPs. Plasmids expressing FLAG-tagged SFV nsPs (nsP1, nsP2, nsP3, and nsP4) were constructed and transfected into 293T cells for western blot (Fig. 6A). As shown in Fig. 6B, all of the FLAG-tagged SFV nsPs were expressed successfully. Then, plasmids expressing ARRDC1 and SFV nsPs were co-transfected into 293T cells for co-immunoprecipitation (co-IP) assay. Interestingly, significant interactions were observed between ARRDC1 and nsP1/3/4, but not nsP2 (Fig. 6C). Considering that the nsP2 protein is predominantly localized in the nuclei when it is expressed alone (26), we further examined the interaction between exogenously expressed nsP2 and ARRDC1 in the SFV-infected cells. As shown in Fig. 6D, the interaction between nsP2 and ARRDC1 was not observed in the absence of viral infection, whereas a weak but specific interaction was detected in the context of viral infection, implying that nsP2 can interact with ARRDC1 in the presence of other nsPs.

Next, we examined whether the interaction between ARRDC1 and nsP1/2/3/4 leads to a degradation of these viral proteins. 293T cells were transfected with plasmids expressing ARRDC1 and individual SFV nsP and harvested at 24 h p.t. for western blot. Surprisingly, when co-expressed with ARRDC1, the protein levels of nsP4 were decreased significantly, whereas the other three nsP levels remained unchanged (Fig. 6E and F). Furthermore, increasing the dosage of the ARRDC1-expressing plasmid resulted in a more significant reduction of nsP4 protein levels (Fig. 6G), indicating that ARRDC1 is involved in the nsP4 protein degradation. To test whether ARRDC1 mediates the degradation of nsP4 through the ubiquitination pathway, we overexpressed SFV nsP4 in 293T cells and then treated the cells with the proteasome inhibitor MG132. The expression levels of SFV nsP4 were remarkably enhanced by MG132 treatment. Conversely, silencing ARRDC1 in 293T cells did not significantly affect SFV nsP4 protein levels, regardless of MG132 treatment (Fig. 6H), suggesting that ARRDC1 regulates the degradation of nsP4 through the ubiquitination pathway.

As the antiviral function of ARRDC1 relies on its cell plasma membrane localization and ubiquitin ligase binding motifs, we further examined the role of these two motifs in the ARRDC1-mediated degradation of nsP4. To this end, we performed a co-IP assay to detect the effects of ARRDC1 mutants (F88L and ΔPPxY) on nsP4 degradation and interaction. The results revealed that both F88L and ΔPPxY mutants exhibited reduced degradation efficiency compared to wild-type ARRDC1 (Fig. 6I, input panel). While the F88L mutant almost lost its binding capacity to nsP4, the ΔPPxY mutant maintained a strong interaction with nsP4, comparable to that of wild-type ARRDC1 (Fig. 6I, IP panel). These findings indicated that ARRDC1-mediated ubiquitination and degradation of nsP4 depend on its cell plasma membrane localization and ubiquitin ligase binding motif. Furthermore, we examined the interaction of ARRDC1-nsP4 in the SFV–infected cells. The results showed that, compared to the mock-infected cells, the interaction between ARRDC1 and nsP4 was significantly enhanced in the context of SFV infection (Fig. 6J), suggesting that the binding of ARRDC1-nsP4 is facilitated by other viral proteins (such as components of the P123 polyprotein) or virus-induced cellular factors.

## DISCUSSION

The arrestin-related trafficking adaptors, such as ARRDC1, ARRDC2, ARRDC3, and ARRDC4, have been identified as anti-viral factors involved in the regulation of virus replication through various mechanisms, such as interacting with HECT ubiquitin ligases and the ESCRT pathway, or activating the innate immune pathway (17, 21, 22). Interestingly, in this study, we revealed a novel mechanism by which ARRDC1 inhibits the replication of SFV through mediating the ubiquitination and degradation of viral nsP4 protein. This effect was observed in a variety of human cell lines, indicating that ARRDC1 is a restriction factor for SFV. It is worth noting that ARRDC1 also exhibits a significant inhibitory effect on SINV, ZIKV, or DENV2 replication, but not HSV-1. Since both SFV, SINV, ZIKV, and DENV2 belong to the positive-strand RNA viruses, the inhibitory effect of ARRDC1 on these viruses suggests that ARRDC1 serves as a potential restriction factor for multiple positive-strand RNA viruses.

Importantly, our data demonstrated that ARRDC1 plays a role in the SFV early replication stage, but not in the attachment and endocytosis processes. As both viral RNA synthesis and nonstructural polyprotein translation occur during the SFV early replication phase, the reduced viral RNA levels in ARRDC1-KO cells at 3 h p.i. could be due to two mechanisms: impaired RNA replication or compromised translation of viral genomic RNA. While our data strongly support the mechanism through ARRDC1-mediated ubiquitination and degradation of nsP4, the potential effect of ARRDC1 on the initial translation of viral RNA remains to be defined. Some dedicated translation assays, such as polysome profiling of viral RNAs or metabolic labeling of viral proteins, would be helpful to evaluate this possibility. Moreover, it is known that the innate immune pathway can be activated during the early stage of viral infection. ARRDC4, another member of the α-arrestins family, has been revealed to recruit TRIM65, thereby promoting K63-linked ubiquitination of MDA5. This activation stimulates the downstream innate immune signaling pathway in response to EV71 infection (22). Therefore, we explored the potential association between the antiviral effect of ARRDC1 and innate immune response. However, although ARRDC1 has a PPxY motif that binds to E3 ubiquitin ligases like ARRDC4, its antiviral effect is independent of the innate immune pathway. The possibility of ARRDC1 recruiting E3 ubiquitin ligases to ubiquitinate proteins in the innate immune signaling pathway requires further investigation.

The release of ARMMs from cell plasma membranes not only depends on its cell plasma membrane localization and ubiquitin ligase binding motifs, but also on the interaction between ARRDC1 and TSG101 (18). Surprisingly, the anti-SFV function of ARRDC1 was not associated with the binding of TSG101, indicating that its antiviral function is independent of the release of ARMMs. Our data also confirmed that both the cell plasma membrane localization and ubiquitin ligase binding motifs of ARRDC1 are required for its anti-SFV capability, suggesting that ARRDC1 functions by anchoring to the cell plasma membrane and subsequently recruiting E3 ubiquitin ligases to exert its antiviral role, a mechanism distinct from that of ARRDC4. These findings were also supported by the co-IP assay between ARRDC1 and nsP4, showing that the binding of ARRDC1-nsP4 depends on the cell plasma membrane localization and ubiquitin ligase binding motifs of ARRDC1. Importantly, we also observed that co-expression of ARRDC1 reduces the nsP4 protein levels in a concentration-responsive manner, which can be restored by the addition of MG132, suggesting that ARRDC1 promotes the degradation of nsP4 via the ubiquitination pathway.

Among SFV nsPs, nsP4 is an RNA-dependent RNA polymerase, mainly involved in the synthesis of nascent viral RNA, which is crucial for the formation of the replication complex (27, 28). The degradation of nsP4 mediated by ARRDC1 results in the inhibition of viral RNA replication, which is consistent with the results from ARRDC1 affecting SFV replication phases that ARRDC1 plays an antiviral role in the early stage of SFV RNA replication. Similarly, recent studies have also shown that host ubiquitin ligases can interact with viral NS proteins, inducing their degradation via the ubiquitination pathway, thereby inhibiting viral replication. For instance, the ubiquitin ligase MARCH8 inhibits influenza A virus infection by targeting viral M2 protein in lysosomes, which contributes to ubiquitination-mediated M2 protein degradation (29). Additionally, both ARRDC1 and ARRDC3 have been reported to interact with YAP1 and facilitate its degradation through E3 ubiquitin ligase Itch-mediated ubiquitination modulation (30). However, the specific E3 ubiquitin ligase through which ARRDC1 exerts its antiviral effect and the function motif(s) involved in the interaction between the ubiquitin ligase and nsP4 remain to be further investigated.

It has been reported that nsP4 is degraded by the ubiquitin-dependent proteolytic pathway in the SINV-infected cells (31), while the underlying mechanism remained unknown for decades. Our work first identified ARRDC1 as the host E3 ubiquitin ligase adaptor specifically targeting nsP4 for ubiquitination and subsequent degradation. Intriguingly, alphaviruses employ a conserved self-limiting strategy through premature termination of nsP4 translation to regulate its replicase activity (31). Our findings further reveal that the host counteracts viral replication by exploiting ARRDC1 to actively degrade nsP4 via the ubiquitination system, thereby adding a previously unrecognized layer of antiviral defense. This dual regulation mechanism (viral self-limitation and host-driven degradation) indicates an evolutionary balance between viral replication efficiency and host immune surveillance.

Interestingly, our study revealed that ARRDC1 binds to another three viral nsPs (nsP1, nsP2, and nsP3), in addition to nsP4. However, these interactions did not pose a significant influence on the expression levels of nsP1/2/3, implying that ARRDC1 may inhibit viral replication through other mechanisms, such as affecting the subcellular localization, activity, or interactions with other host factors of nsP1/2/3. As an enzyme responsible for mRNA capping, nsP1 is involved in the methylation and capping of viral mRNA, which is essential for viral replication and translation (27, 32). nsP2 is a multifunctional protein with several crucial roles in the SFV life cycle, including processing the nonstructural polyproteins and recruiting other viral and host factors necessary for viral RNA synthesis (33, 34). nsP3, while its precise function remains elusive, plays an important role in recruiting host factors for the assembly of the viral replication complex (35). Considering the roles of nsP1, nsP2, and nsP3 in viral replication, the interactions between ARRDC1 and these proteins may affect the synthesis of viral RNA and the stability of the replication complex. These interactions could be a host defense mechanism that limits viral replication by disrupting the formation or function of the viral replication complex. Given the interaction between nsP1/2/3 and ARRDC1, a potential binding competition between nsP1/2/3 and nsP4 for ARRDC1 may exist. If high levels of nsP1, nsP2, or nsP3 accumulate, they could reduce ARRDC1’s availability to interact with nsP4, which might preserve nsP4 levels and promote viral replication. This dynamic could represent a previously uncharacterized regulatory mechanism in virus-host interactions. Future experiments could test this hypothesis by overexpressing nsP1/2/3 and monitoring nsP4 stability and viral replication levels.

To be pointed out, because there are no commercial antibodies against SFV nsPs available, we alternatively used FLAG-tagged nsPs to examine their interactions with ARRDC1. Although epitope tags might potentially disrupt alphavirus nsPs localization, interaction interfaces, or normal function, our co-IP data obtained from the virus-infected cells and functional validations (such as ARRDC1-knockout affecting viral replication) supported the biological relevance of these interactions. To rule out the potential tagging artifacts, future studies will employ ubiquitin fusion systems to eliminate artificial N-terminal methionine residues and confirm these interactions in tag-free systems.

In summary, we identified ARRDC1 as a novel restriction factor of SFV replication and elucidated the molecular mechanism of its antiviral function: Upon SFV infection, ARRDC1 affects the formation of the viral replication complex through interacting with nsP1, nsP2, nsP3, and nsP4, facilitating the ubiquitination and degradation of nsP4, eventually suppressing the SFV replication. Further investigation on the intricate mechanisms of ARRDC1-nsP1/2/3/4 interactions will be helpful for understanding the interplay of host-SFV interaction and also provide potential targets for anti-SFV therapeutics.

## MATERIALS AND METHODS

### Cell culture

Human hepatoma cells (Huh7) were provided by Professor Yiping Li from Sun Yat-sen University. Huh7, human lung carcinoma epithelial cells (A549, ATCC CCL-185), human cervical cancer cells (HeLa, ATCC CCL-2), and human embryonic kidney cells (293T, ATCC CRL-3216) were cultured in Dulbecco’s modified Eagle medium (DMEM, Sigma) supplemented with 10% fetal bovine serum (FBS, Sigma) at 37°C with 5% CO_2_. African green monkey kidney cells (Vero, ATCC CCL-81) and baby hamster kidney cells (BHK-21, ATCC, CCL-10) were maintained in DMEM supplemented with 5% FBS. Media were supplemented with 100 U/mL of streptomycin and penicillin (Invitrogen).

### Antibodies

Primary antibodies used in this study included anti-ARRDC1 (ab181758, Abcam), anti-FLAG (PM020, MBL), anti-HA (M180-3, MBL), and anti-GAPDH (10494-1-AP, Proteintech). Anti-SFV E2 was prepared by Hangzhou HUABIO Company. Secondary antibodies included IRDye 800 CW-conjugated anti-rabbit IgG (926-3221, LI-COR) and IRDye 680 CW-conjugated anti-mouse IgG (926-68020, LI-COR). The secondary antibody used in the immunofluorescence assay included goat anti-rabbit IgG secondary antibody (Alexa Fluor 488, Invitrogen).

### Virus

Semliki Forest virus (SFV, strain 4) was kindly provided by Professor Xi Zhou from Wuhan Institute of Virology, Chinese Academy of Sciences. Sindbis virus (SINV TR339-GFP, derived from the TR339 strain) was constructed by inserting a GFP gene followed by the Thosea asigna virus 2A self-cleaving peptide sequence between the capsid and PE2 genes in the SINV TR339 molecular clone (kindly provided by Professor Rong Zhang from the School of Basic Medicine of Fudan University) (36, 37). Zika virus (ZIKV, H/PF/2013 strain) and dengue virus type 2 (DENV2, 16681 strain) were donated by the Guangzhou Municipal Center for Disease Control and Prevention. All viruses are kept in the −80°C refrigerator.

### Viral infection

Cells were infected with SFV at an MOI of 0.1 or 1, whereas they were infected with SINV, ZIKV, and DENV at an MOI of 1. According to the requirements of the experiments, viral supernatants were harvested at indicated time points, and cell debris was removed by centrifugation at 200 × *g* for 5  min and filtration, followed by real-time PCR, western blot, plaque assay, or other detections. The green fluorescence fused with SINV could be visualized by an inverted fluorescence microscope (DMi8, Leica Microscopes, Wetzlar, GER).

### Virus titration

Standard plaque assay was used to detect virus titers. Vero or BHK21 cells were inoculated in a 12-well or 24-well plate. The samples were prepared in serial 10-fold dilutions, and 100 µL/well of the diluted virus was added. The cells were incubated with the diluted virus at 37 °C for 1 h. Then the medium was removed and cultured in 2% methylcellulose (1:1) (Sigma-Aldrich) or the mixture of 2× MEM (Invitrogen) and isopycnic 2% low melting point agarose (Sangon Biotech) (1:1). Visible plaques were counted at 1 to 2 days (SFV and SINV) or 4–7 days (ZIKV and DENV2). A 10% formaldehyde solution was used to fix cells, and plaques were visualized by staining with 1% crystal violet.

### siRNA synthesis and interference

The negative control siRNA (siNC) and the siRNAs targeting ARRDC1 were synthesized by Tsingke Biotechnology Company (Beijing, China). The siRNA sequences are listed in Table S1. Transient transfection of siRNAs (20 nmol) was performed using Lipofectamine 2000 reagent (Invitrogen) according to the manufacturer’s instructions.

### Quantitative reverse transcription PCR

Total RNAs were extracted using TRIzol reagent (Invitrogen). RNAs were reverse transcribed using HI Script Q RT SuperMix (Vazyme) and then amplified by PCR using SYBR Select Master Mix (Vazyme) on the CFX96 Real-Time Detection System (Bio-Rad). Primers used for qRT-PCR are listed in Table S2. The ΔΔCt method was used to analyze the qRT-PCR data.

### Western blotting

Cells were lysed with lysis buffer (50 mM Tris-HCl [pH 7.4], 150 mM NaCl, 0.5% [vol/vol] NP-40, 1% Triton X-100, 1 mM EDTA, 1% protease inhibitor mixtures, and 1 mM PMSF). Then, the lysates were boiled in loading buffer (200 mM Tris-HCl [pH 6.8], 50 mM EDTA, 8% SDS, 0.08% bromophenol blue, 5% [vol/vol] β-mercaptoethanol, 40% [vol/vol] glycerin) for 10 min. Proteins were separated on SDS-PAGE, then transferred onto nitrocellulose membranes. Membranes were blocked with 5% BSA in 0.1% PBST. Primary antibodies diluted in 0.1% TBST with 3% BSA were incubated at 4°C overnight, and secondary antibodies were incubated at room temperature for 1 h. Odyssey IR imaging system (LI-COR) was used for detection, and Image Lab (Bio-Rad) was used to quantify the western blot results.

### Plasmid construction

Oligonucleotide sequences of single guide RNAs targeting human ARRDC1 are listed in Table S3. Oligonucleotides were annealed and inserted into the plasmid vector lentiCRISPR v2 (Addgene, #52961). Positive plasmids were designated as pLenti-sgARRDC1#1 and pLenti-sgARRDC1#2.

Fragments of full-length ARRDC1 and its mutants were amplified by PCR using ARRDC1 cDNA template. Primer sequences are listed in Table S4. PCR fragments were purified and cloned into lentiviral vector pLV-EF1α-IRES-blasticidin (Addgene, #85133). The HA tag was fused to the C-termini of full-length ARRDC1 and its mutants. Positive clones were verified by DNA sequencing.

Coding sequences for SFV nsPs (nsP1, nsP2, nsP3, and nsP4) were amplified by PCR from viral cDNA and cloned into pLV-EF1α-IRES-blasticidin plasmid: nsP1/nsP3 at EcoRI/NotI sites, nsP2 at BamHI/NotI sites, and nsP4 at BamHI/EcoRI sites. For nsP1/nsP2/nsP3, the nucleotide sequence encoding the FLAG-tag was added to the 3′ end of their PCR products, fusing the FLAG-tag to their C-termini upon expression. For nsP4, the FLAG-tag-encoding sequence was incorporated at the 5′ end of its PCR-amplified coding region, leading to N-terminal FLAG-tag fusion of the expressed protein. Sequences of primers are listed in Table S4.

### Generation of ARRDC1-knockout cells by CRISPR/Cas9 gene editing

The sgRNA vector (pLenti-sgARRDC1#1 and pLenti-sg ARRDC1#2) and two packaging plasmids, pSPAX2 (Addgene, #12260) and pVSVG (Addgene, #12259), were transfected into 293T cells using Lipofectamine 2000 reagent (Thermo). Lentivirus supernatants were collected at 48 h p.t. and transduced into A549 cells. One day later, cells were transferred to new cell culture plates and selected by puromycin (1 µg/mL) for more than 7 days to isolate single-cell clones.

Then, the cell clones were confirmed by genomic DNA extraction and sequencing. DNA sequencing results revealed that there were three distinct frameshift deletion variants in ARRDC1-KO1 cells (exon 1-edited): 26, 10, and 152 bp deletions (Fig. 2B, top panel). These mutations led to premature stop codons at amino acid positions 37, 40, and 54, respectively, predicted to produce a less than 6 kDa truncated peptide lacking all known functional domains (including the arrestin and PPxY motifs). In addition, there were three distinct frameshift deletion variants in ARRDC1-KO2 cells (exon 6-edited): 2, 4, and 10 bp deletions (Fig. 2B, bottom panel). These mutations introduced premature stop codons at amino acid positions 317, 267, and 265, respectively, predicted to produce a 29–35 kDa truncated peptide lacking the PPxY motif.

While the western blot data showed weak bands near full-length ARRDC1 size (47.6 kDa) in KO clones (Fig. 2A), it is noted that for the ARRDC1-KO1 clone, it is predicted to produce only <6 kDa truncated peptide, which is far below the detection range of standard western blot (~10 kDa); for the ARRDC1-KO2 clone (exon 6-edited), it could theoretically encode 29–35 kDa truncated proteins. However, the commercial anti-ARRDC1 antibody (ab181758, Abcam) was raised against an undisclosed epitope that may not recognize these truncated forms. Although minimal truncated protein production cannot be formally excluded, the DNA sequencing data demonstrated effective deletion of functional ARRDC1. This is because all PPxY motifs (aa 402–405 and aa 415–418, critical for ubiquitin ligase binding function) are absent in both KO1 and KO2 variants. Since the ARRDC1 protein is difficult to detect and a high antibody concentration (1:500 dilution) may result in elevated background signals, the residual bands in Fig. 2A likely represent nonspecific detection rather than intact or truncated ARRDC1 protein.

### Virus entry assay

In the virus binding assay, control and knockout cells were pre-chilled on ice before SFV infection. Cells were washed three times with ice-cold PBS and infected with SFV at an MOI of 3 with 2% FBS DMEM, followed by incubation on ice for 1 h. For the virus internalization assay, cells were infected at 37°C for 30 min. After three washes with PBS buffer, cells were lysed in TRIzol reagent. Total RNAs were extracted to detect viral RNAs, which were normalized to *β-actin* mRNA levels.

### Immunofluorescence and confocal microscopy

Cells were infected with SFV at an MOI of 3. At 12 h p.t., cells were washed three times with PBS buffer and fixed with 4% PFA for 20 min. Next, the cells were permeabilized for 15 min using 0.02% Triton-X 100 and blocked with 5% BSA for 1 h. Then the cells were incubated with the primary antibody at 4°C overnight. After another three times of wash with PBS buffer, cells were incubated with Alexa Fluor 488-conjugated anti-rabbit IgG (Invitrogen) in PBS buffer at room temperature for 1 h, followed by incubation with DAPI (Invitrogen) diluted in PBS buffer for 20 min. Fluorescence images were acquired with the Nikon C2 microscope and analyzed with the NIS Elements software.

### Co-IP assay

293T cells were co-transfected with expression plasmids of ARRDC1 and SFV nsPs by Lipofectamine 2000 reagent. At 48 h p.t., cells were harvested with ice-cold RIPA lysis buffer (50 mM Tris-HCl [pH 7.4], 150 mM NaCl, 0.5% [vol/vol] NP-40, 1% Triton X-100, 1 mM EDTA, 1% protease inhibitor mixtures, 1 mM PMSF, 1 mM Na_3_VO_4_, and 1 mM NaF). Twenty percent of the total cell lysate was reserved as an input control. Eighty percent of the total cell lysate was incubated with anti-HA agarose beads (HNA-25-500, NuoyiBio) at 4°C overnight. Beads were washed several times, and bound proteins were eluted by boiling in loading buffer for western blot analysis.

### Treatment of MG132

MG132 (HY-13259, MCE) was dissolved in DMSO at a stock concentration of 40 mM. Cells were transfected with siRNA for 36 h, followed by transfection with plasmids for another 24 h. Before harvesting, cells were incubated with media containing DMSO or MG132 (10 µM) for 1 h.

### Statistical analysis

Data were analyzed by GraphPad Prism 10.0 software. All the statistical analyses were performed using an unpaired two-tailed Student’s *t*-test, or analysis of variance (ANOVA) with Dunnett’s multiple comparison test. All the data were shown as means ± standard error of the mean (SEM), the data points and bar graphs represented the mean of independent biological replicates. The differences were considered statistically significant at *P* < 0.05.

## Data Availability

All data are available in the main text and the supplemental material.

## SUPPLEMENTAL MATERIAL

Supplemental tables jvi.00977-25-s0001.docx Tables S1 to S4.

ASM does not own the copyrights to Supplemental Material that may be linked to, or accessed through, an article. The authors have granted ASM a non-exclusive, world-wide license to publish the Supplemental Material files. Please contact the corresponding author directly for reuse.
