# Supplementary material for: ARRDC1 inhibits the replication of Semliki Forest virus by regulating the ubiquitination and degradation of viral nsP4
Source: J Virol. 2025 Aug 18;99(9):e00977-25. doi: 10.1128/jvi.00977-25 (PMC12455954; doi:10.1128/jvi.00977-25)
Supplement: Supplemental tables — Tables S1 to S4. [file jvi.00977-25-s0001.docx]

## Supplemental material

## Table S1. Oligonucleotides of siRNA

| Gene | Sequence (5´- 3´) |
| --- | --- |
| *ARRDC1*  siRNA | 5F: CAGCCUCGUGUUCUAUAUCUU |
|  | 3R: AAGAUAUAGAACACGAGGCUG |

## Table S2. Sequences of primers used in qRT-PCR

| Gene | Sequence (5´- 3´) |
| --- | --- |
| *ARRDC1* | 5F: CTTCCGAGCTATCCGGGTGA  3R: GTCCCTCAAAAGATGTGGGTG |
| *IFN-β* | 5F: AAACTCATGAGCAGTCTGCA  3R: AGGAGATCTTCAGTTTCGGAGG |
| *MX1* | 5F: GCACACACCCAACTGTCAGCGA  3R: CCCATGTCCGAAACTCTCTGCGG |
| SFV | 5F: CCGGAGGACGCACAGAAGTTG  3R: TGCGACGGCCACAATCGGAAG |
| SINV | 5F: CATCGGTGAGAGACCACCTT  3R: AACCACGCCTTTGTTTCATC |
| *β-actin* | 5F: GCTCCTCCTGAGCGCAAG  3R: CATCTGCTGGAAGGTGGACA |

## Table S3. Oligonucleotides of ARRDC1-sgRNA

| Gene | Sequence (5´- 3´) |
| --- | --- |
| *ARRDC1* sgRNA-1 | 5F: CACCGGCACCGCTGCCGTTCCGAGG |
|  | 3R: AAACCCTCGGAACGGCAGCGGTGCC |
| *ARRDC1* sgRNA-2 | 5F: CACCGCGACGTACGGACCATTGCGG |
|  | 3R: AAACCCGCAATGGTCCGTACGTCGC |

## Table S4. Sequences of primers used in PCR amplification of gene fragments

| Gene | Sequence (5´- 3´) |
| --- | --- |
| *ARRDC1* | 5F: CGGAATTCATGGGGCGAGTGCAGCTCTT  3R: TTGCGGCCGCTCAGCTCTCAGGGGTCAGGCTG |
| *ARRDC1*-RES | 5F: AGTATGTAAAAGACCAAAGAACACTTGTGATCCTTGGAAA  3R: TTCTTTGGTCTTTTACATACTGAGCCCCTTGAACCTGAAC |
| *ARRDC1*-HA | 5F: CGGAATTCATGGGGCGAGTGCAGCTCTT  3R: TTGCGGCCGCTCAAGCGTAATCTGGAACATCGTAT  GGGTAACCACCACCGCTCTCAGGGGTCAGGCTG |
| △N191 | 5F: CGGAATTCATGCAGTCAGGCAAGGACACCAG  3R: TTGCGGCCGCTCAAGCGTAATCTGGAACATCGTAT  GGGTAACCACCACCGCTCTCAGGGGTCAGGCTG |
| F88L | 5F: CACAGCTTCCCCTTACAGTTCC  3R: GGAACTGTAAGGGGAAGCTGTG |
| PAAP | 5F: TGGTGGTGCCTGCCGCACCA  3R: TGGTGCGGCAGGCACCACCA |
| △PPEY | 5F: CGGAATTCATGGGGCGAGTGCAGCTCTT  3R: GGCCTCATAGGGGTAGCCCCAAGAACTAAGAATCA  AGGTGCTGG |
| △PPSY | 5F: CGGAATTCATGGGGCGAGTGCAGCTCTT  3R: GCTCTCAGGGGTCAGGCTGGGTTCCACGCCGCCGCAGCTCTGCTCGGCCTCATAGGGGTAGCCCCAAGAACT |
| SFV nsP1 | 5F: CGGAATTCGCCACCATGGCCGCCAAAGTGCATGTTG  ATAT  3R: TTGCGGCCGCTCACTTATCGTCGTCATCCTTGTAATCACCACCACCTGCACCTGCGTGATACTCTAGTTC |
| SFV nsP2 | 5F: CGGGATCCGCCACCATGGGGGTCGTGGAAACACCTCGCA  3R: TTGCGGCCGCTCACTTATCGTCGTCATCCTTGTAATCACCACCACCACACCCGGCCGTGTGCATGGCTTCTC |
| SFV nsP3 | 5F: CGGAATTCGCCACCATGGCACCATCCTACAGAGTTAAGAGAGC  3R: TTGCGGCCGCTCACTTATCGTCGTCATCCTTGTAATCACCACCACCTGCACCCGCGCGGCCTAGTCGCAGGAC |
| SFV nsP4 | 5F: CGGGATCCGCCACCATGGATTACAAGGATGACGAC GATAAGGGTGGTGGTTATATTTTCTCCTCGGACACTGGCA  3R: CGGAATTCTCAACGCACCAATCTAGGACCGCCGTA GAG |
